# Judicious Use of Ozanimod for Ulcerative Colitis and Multiple Sclerosis

**DOI:** 10.14309/crj.0000000000001332

**Published:** 2024-04-15

**Authors:** Gabriela F. Jhon, Erin M. Forster

**Affiliations:** 1 Department of Internal Medicine, Grand Strand Medical Center, Myrtle Beach, SC; 2 Division of Gastroenterology and Hepatology, Medical University of South Carolina, Charleston, SC

**Keywords:** Ozanimod, ulcerative colitis, inflammatory bowel disease, multiple sclerosis, sphingosine-1-phosphate receptor modulator

## Abstract

Clinical trials have demonstrated the efficacy of ozanimod, an oral sphingosine-1-phosphate receptor modulator, for the treatment of moderate-to-severe ulcerative colitis. Infrequently does an opportunity present itself to use one drug for two simultaneous disease states, proving especially beneficial in the case of this patient intolerant of numerous established therapies for ulcerative colitis. This case report describes the successful use of ozanimod for both ulcerative colitis and multiple sclerosis, achieving clinical remission in both diseases.

## INTRODUCTION

Recent clinical trials have demonstrated the efficacy of ozanimod, a once-daily oral sphingosine-1-phosphate (S1P) receptor modulator, for the treatment of moderate-to-severe ulcerative colitis.^1^ S1P receptor modulators attenuate the immune response by reducing circulating lymphocytes and targeting subtypes of T lymphocytes that have shown to play a role in autoimmune diseases. This reduction in the migration of lymphocytes to the intestinal mucosa reduces areas of colonic inflammation.

Data from clinical trials indicate 10 weeks of ozanimod use provided symptomatic relief in 48% of patients with 18% achieving remission, whereas 1 year of use provided symptomatic relief to 60% of patients with 37% achieving remission. Improvement in symptoms, such as decreased rectal bleeding and stool frequency, was observed as early as 2 weeks after initiating treatment.^2^

The S1P receptor modulators also offer beneficial effects such as neuroprotection and neuromodulation by targeting several pathophysiological pathways also implicated in multiple sclerosis, leading to ozanimod's approval for such in 2020.^2^ This case report describes the successful use of ozanimod for both ulcerative colitis and multiple sclerosis, achieving clinical remission in both diseases.

## CASE REPORT

A 52-year-old white woman with a medical history of psoriasis was diagnosed with ulcerative colitis at an outside facility after presenting with abdominal pain and bleeding; colonoscopy at the time demonstrated severe left-sided inflammation and pathology consistent with chronic active colitis. Initial therapy included mesalamine and intermittent steroids. Several months later, the patient stopped taking mesalamine because of a loss of health insurance. The patient reported being symptomatically controlled without therapy for about 10 years before experiencing a return in her symptoms when she stopped smoking cigarettes. She established care with our facility's inflammatory bowel disease clinic and was started on a regimen of adalimumab and mesalamine. About 5 months later, adalimumab was discontinued after reporting excessive fatigue and “low quality of life.” She then began vedolizumab along with mesalamine. On this regimen, the patient showed initial endoscopic improvement, but symptoms worsened over the next year and a half, despite consistent uptitration of vedolizumab to maximum dosage and the addition of methotrexate. At this time, repeat endoscopic evaluation revealed Mayo 3 colitis with spontaneous bleeding and ulcerations. This was deemed a failure of vedolizumab treatment, and the patient was promptly switched to infliximab.

High-dose accelerated infliximab (10 mg/kg every 4 weeks) in combination with methotrexate and high-dose mesalamine by mouth (PO) finally allowed the patient to achieve symptomatic, endoscopic, and histologic remission. Patchy, Mayo 1 pancolitis was seen on colonoscopy after 1 year on this regimen, demonstrating a significant improvement from her previous study. Methotrexate was stopped 3 months after this endoscopic evaluation because of the patient reporting headache and “brain fog.” Only months after remission was achieved, the patient developed lower extremity tingling and right foot drop and was diagnosed with demyelinating disease on magnetic resonance imaging (MRI). Notably, on further questioning, she did endorse central nervous system deficits dating back more than 10 years, starting with optic neuritis. The patient reports undergoing a previous MRI which was remarkable for one unspecified lesion, but she did not receive an official diagnosis.

After shared decision-making and multidisciplinary discussions between the patient's neurology and gastroenterology teams, infliximab was discontinued because of the increased risk of further demyelination. The decision was made to start ozanimod therapy. Since the initiation of ozanimod, the patient's neurologic deficits have not progressed, gastrointestinal (GI)-related symptoms are under control, and colonoscopy after 6 months of therapy demonstrated only mild Mayo 1 left-sided disease. Repeat colonoscopy after 18 months of therapy demonstrated Mayo 0-1 disease with biopsies showing minimal active colitis. erythrocyte sedimentation rate (ESR) and C-reactive protein (CRP) are within normal limits. Her latest MRI was negative for any active demyelinating lesions.

The patient's current maintenance regimen is ozanimod 0.92 mg daily and mesalamine 4.8 g daily, which she is tolerating well with no side effects. She has graduated from physical therapy and is back to jogging 2 miles three times per week.

## DISCUSSION

Screening for neurologic diseases such as multiple sclerosis influences drug selection in patients with inflammatory bowel disease (IBD), precluding use of medications like anti-TNFs. The increased risk of progressive multifocal leukoencephalopathy (PML) has led to medications such as natalizumab to require intensive prescribing programs to ensure appropriate training for prescribers. Although there have been reported cases of PML in patients with multiple sclerosis treated with S1P receptor modulators, only one case has been reported with the use of ozanimod among the 5,180 patients who had received the drug during its clinical trials.^3^ The incidence of PML seems to be low overall in patients taking S1P receptor modulators, allowing it to persist as an effective choice for patients intolerant to other therapies and/or in need of treatment for two disease states such as in the case of our patient. In addition to neurologic side effects, ozanimod has also been associated with instances of elevated liver aminotransferase levels, although this did not meet criteria suggestive of drug-induced liver injury. Bradycardia was noted in patients taking ozanimod therapy in early trials when compared with placebo; however, no cases of advanced heart block resulted from this.^2^ Use of an S1P receptor modulator such as ozanimod has a unique potential to treat ulcerative colitis and multiple sclerosis, whose respective therapies are often contraindicated when they are comorbid diseases. Infrequently does an opportunity present itself to use one drug for both disease states, proving especially beneficial in the case of this patient intolerant of numerous established therapies for ulcerative colitis.

## DISCLOSURES

Author contributions: GF Jhon and EM Forster substantial contribution to the conception of the work, drafting and revision of the work, final approval of the version to be published, and agreement to be accountable for all aspects of the work. EM Forster is the article guarantor.

Financial disclosure: None to report.

Previous presentation: Advances in Inflammatory Bowel Disease; December 5, 2022; Orlando, Florida.

Informed consent was obtained for this case report.
